# Status epilepticus in patients with genetic generalized epilepsy: a case series study

**DOI:** 10.1186/s42494-023-00144-1

**Published:** 2023-12-14

**Authors:** Gengyao Hu, Bi Wang, Beibei Chen, Zezhi Wang, Ze Chen, Yonghong Liu

**Affiliations:** Department of Neurology, Xijing hospital, Fourth Military Medical University (Air Force Medical University), Xi’an, 710032 PR China

**Keywords:** Genetic generalized epilepsy, Status epilepticus, Electroencephalography

## Abstract

**Background:**

Genetic generalized epilepsy (GGE) accounts for nearly one-third of all epilepsies. The feature of status epilepticus (SE) in patients with GGE has been rarely studied. We aimed to determine the electroclinical characteristics of SE in patients with GGE.

**Methods:**

In this retrospective study, nine patients with GGE were enrolled at Xijing Hospital, Xi’an, China from May 2014 to May 2020. SE was confirmed by 24-h video-EEG recording. The demography, clinical manifestation, brain MRI and SE pattern were analyzed.

**Results:**

Of the nine patients in the study, seven were female. The mean age of the patients at the time of inclusion was 16.8 years (range 7–31 years), and the mean age at the onset of epilepsy was 10.9 years (range 6–17 years). The follow-up time ranged from 3 months to 6 years. Myoclonic absence status was identified in four patients showing eyelid myoclonia with absence and one patient showing perioral myoclonia with absences. Myoclonic SE was identified in three patients showing juvenile myoclonic epilepsy. Autonomic SE was found in one patient with eyelid myoclonia with absence. SE was terminated by oral midazolam in four patients. In the other five patients, SE terminated spontaneously.

**Conclusions:**

The seizure type of SE in patients with GGE is often consistent with their major symptoms. Oral midazolam may be an option to terminate SE in patients with GGE.

## Background

Status epilepticus (SE) is a common neurological emergency with high morbidity and mortality [1–3]. In 2015, the International League Against Epilepsy proposed a new definition of SE: a condition resulting either from the failure of the mechanisms responsible for seizure termination or from the initiation of mechanisms that lead to abnormally prolonged seizures (after time point t1) [4]. SE is divided into convulsive SE, myoclonic SE, myoclonic absence status and other forms, according to the presence or absence of prominent motor symptoms. The t1 times for initial treatment of bilateral convulsive SE, focal SE with impaired consciousness and absence SE are 5 min, 10 min and 10–15 min respectively. However, the ILAE has not provided a precise definition of t1 time for other SE types.

Genetic generalized epilepsy (GGE) is a broad group of epilepsies with generalized seizure types and generalized spikewave, which includes idiopathic generalized epilepsy (IGE) and other generalized epilepsies that may be genetically related [5–7]. Juvenile myoclonic epilepsy (JME), epilepsy of eyelid myoclonia with absences (ELMA), and perioral myoclonia with absences (POMA) are currently recognized as GGE [6, 8, 9]. GGE can display various types of SE, such as myoclonic SE, myoclonic absence status, and autonomic SE. However, the precise presentation of SE in GGE patients as well as their response to drugs remains largely unclear. In this study, we set out to assess the electroclinical features, treatment, and follow-up of SE in a cohort of Chinese patients with GGE.

## Materials and methods

### Participants

This study was designed as a retrospective study. We searched the EEG database at our epilepsy center, Department of Neurology, Xijing Hospital (Xi’an, China) using keywords “SE, GGE, IGE” for corresponding patients from May 2014 to May 2020. The video-EEG reports of these patients were reviewed. This research was approved by the Medical Ethics Committee of the First Affiliated Hospital of the Air Force Mdical University (KY20222046-C-1) and written informed consent was obtained from all participants.

### Patient inclusion criteria

The inclusion criteria for patients with GGE were as follows: (1) onset of initial seizure before age 20; (2) normal neurological examination during interictal periods; (3) generalized ictal polyspike/spike-wave discharges and normal background EEG; (4) normal brain magnetic resonance imaging (MRI) [10].

The definition of SE was adopted from ILAE: a seizure that shows no clinical signs of arresting after a duration encompassing the great majority of seizures of that type in most patients or recurrent seizures without interictal resumption of baseline central nervous system function [11].

### Collection and follow-up of clinical data

Clinical data of GGE/SE cases were collected, including age, sex, MRI, Wechsler IQ, age at first seizure onset, type of first seizure, specific type of GGE, clinical-EEG characteristics of SE, and medical treatment before, during and after SE. A follow-up procedure was conducted.

## Results

### Cohort characteristics

SE was observed in nine patients with GGE, including seven females and two males. The mean age at the time of inclusion in our study was 16.8 years (range 7–31 years). The mean age at seizure onset was 10.9 years (range 6–17 years). The follow-up time ranged from 3 months to 6 years. Of the nine patients, five were diagnosed with ELMA, three with JME, and one with POMA epilepsy. Normal background EEG activity and interictal generalized spike-and-wave discharges were observed in all patients. In addition, all patients in the study had normal IQ at the time of their EEG recording, and the results of brain MRI were negative.

### SE

A total of 10 episodes of generalized SE were identified in the nine patients during EEG monitoring. Myoclonic absence status was reported in four patients with ELMA and one patient with POMA; myoclonic SE was reported in three patients with JME; and autonomic SE in one patient with epilepsy of eyelid myoclonia. The electroclinical characteristics of the patients are summarized in Table 1.

**Table 1:** Electroclinical characteristics of GGE patients with presentation of SE

Case (age, sex)	GGE syndrome	Age of onset in years, type of first seizure	Clinical presentation of SE	EEG characteristics (duration)	Possible cause of SE	ASMs before SE	Treatment at SE	Treatment after SE	Outcome and follow-up time
1(8y, F)	EMA epilepsy	6 years, EM	Eyelid myoclonus, consciousness impairment, occasional limb myoclonus, head jerk	3–5 Hz generalized poly/spike-wave discharges (120 min)	No ASMs	None	MDZ	VPA (0.75 g/day)	Seizure-free (3years)
2(10y, F)	EMA epilepsy	10 years, GTCS	Increased heart rate, occasional eyelid myoclonus	Generalized slow waves admixed with spikes waves (twice, 15 and 19 min, respectively)	Ill-advised ASMs (OXC)	OXC (0.3 g/day)	None	LEV (1 g/day)	Seizure-free (0.25 year)
3(19Y, F)	EMA epilepsy	13 years, GTCS	Eyelid myoclonus, consciousness impairment, secondary GTCS	3–5 Hz generalized poly/spike-wave discharges (46 min)	Ill-advised ASMs (OXC)	OXC (0.6 g/day)	MDZ	VPA (0.5 g/day) LEV (1.5 g/day)	Seizure-free (2 years)
4(22y, F)	EMA epilepsy	8 years, EMA	Eyelid myoclonus, consciousness impairment, occasional limb myoclonus, head jerk	Paroxysmal 2.5–3 Hz generalized polyspike/spike-wave discharges (60 min)	Insufficient ASMs (LTG)	LTG (0.025 g/day)	MDZ	LEV (1 g/day) TPM (0.15 g/day)	Infrequent seizures (1 year)
5(24y, F)	EMA epilepsy	15 years, GTCS	Eyelid myoclonus, slight limb myoclonus, consciousness impairment, secondary GTCS	Generalized polyspike/spike-wave discharges (180 min)	Ill-advised ASMs (CBZ)	VPA (1 g/day) CBZ (0.6 g/day)	None	VPA (1 g/day) TPM (0.2 g/day)	Seizure-free (2 years)
6(12y, M)	JME	8 years, MJ	Limb myoclonus, consciousness impairment, secondary GTCS	Irregular generalized slow waves admixed with polyspike/spike-wave discharges (35 min)	No ASMs	None	None	VPA (0.5 g/day)	Seizure-free (2 years)
7(18y, F)	JME	15 years, MJ	Limb myoclonus	Paroxysmal 3–4 Hz generalized polyspike/spike-wave discharges (180 min)	Insufficient ASMs (VPA)	VPA (1 g/day)	MDZ	VPA (1.5 g/d)	Seizure-free (6 years)
8(31y, F)	JME	17 years, GTCS	Limb myoclonus, secondary GTCS	Paroxysmal 3–4 Hz generalized polyspike/spike-wave discharges (30 min)	No ASMs	None	None	LTG (0.05 g/d)	Frequent seizures (5 years)
9(7, M)	POMA epilepsy	6 years, PMA	Perioral myoclonus, consciousness impairment	2.5–3 Hz generalized spike-wave discharges (20 s)	No ASMs	None	None	VPA (0.5 g/d)	Seizure free (2 years)

*Abbreviations*: *F *Female, *M *Male, *GGE *Genetic generalized epilepsy, *SE *Status epilepticus, *EMA *Eyelid myoclonia with absences, *JME *Juvenile myoclonic epilepsy, *POMA *Perioral myoclonia with absences, *GTCS *Generalized tonic-clonic seizure, *EM *Eyelid myoclonia, *MJ *Myoclonic jerks, *ASMs *Antiseizure medications, *OXC *Oxcarbazepine, *LTG *Lamotrigine, *VPA *Valproate, *LEV *Levetiracetam, *TPM *Topiramate

**Fig. 1:**
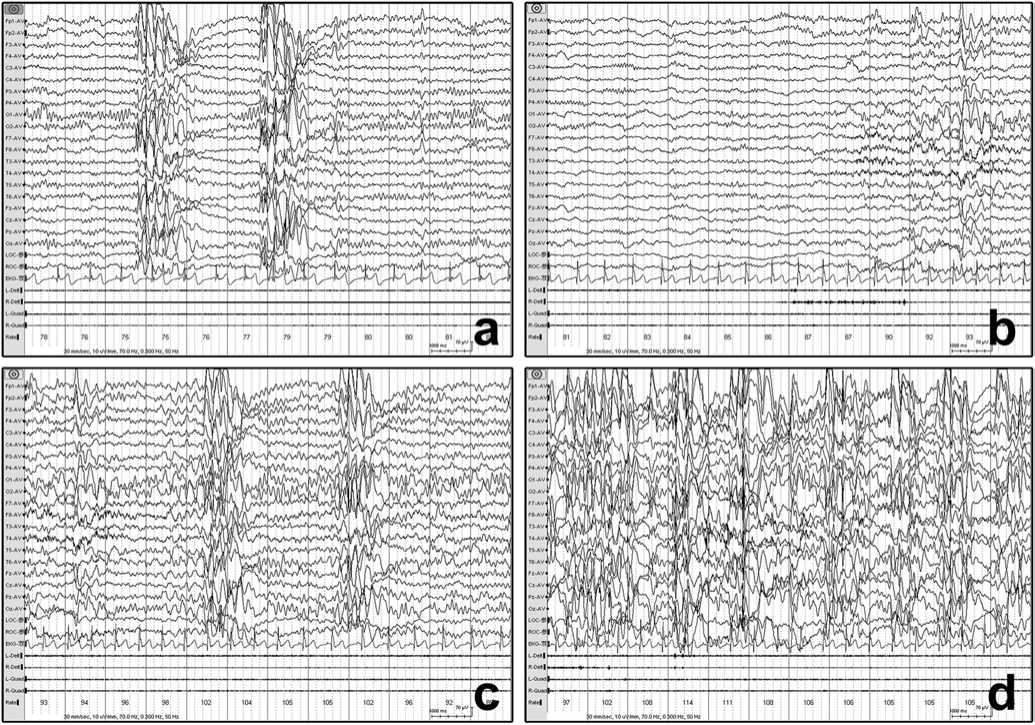
Example of the electroclinical characteristics of patient 2 with autonomic SE. **a** Interictal EEG showed paroxysmal generalized polyspike-wave discharges with a baseline heart rate of 80 beats/min without any clinical symptoms. **b**-**d**. Continuous EEG showed increased generalized polyspike-wave discharges and heart rate ranging from 80 to 110 beats/min associated with eyelid fluttering after arousal. L-Delt: left deltoid, R-Delt: right deltoid, L-Orbi: left orbicularis oculi, R-Orbi: right orbicularis oculi

The clinical manifestation of eyelid myoclonic absence status was characterized by eyelid jerks with mild consciousness impairment and occasional jerks of the head and limbs. These episodes and synchronous epileptiform discharges (EDs) of generalized spike-wave discharges occurred immediately after the eyes were closed, with a duration varying from 15 to 350 min. Interestingly, Patient 2 with ELMA experienced prolonged increase of heart rate (80 to 110 beats/min) and eyelid fluttering without consciousness impairment; the episode occurred twice, lasting for 15 and 19 min, respectively. Synchronous EEG showed generalized slow waves admixed with spikes waves (Fig. 1).

Three patients with JME developed myoclonic SE. Patient 6 presented with myoclonic jerks of the upper limb with consciousness impairment. Ictal EEG showed irregular slow waves admixed with polyspike/spike-wave discharges, and the EMG recording showed that the burst activity appeared in clusters, each lasting for 300 to 500 ms; the episode lasted for 35 min. The other two patients had myoclonic jerks of unilateral or bilateral upper limbs without consciousness impairment, and the ictal EEG showed paroxysmal 3 to 4 Hz polyspike/spike-wave discharges, which lasted 30 to 180 min.

A series of perioral myoclonus accompanied by impaired consciousness were recorded in the POMA case. Ictal EEG showed 2.5 to 3 Hz spike-wave discharges, and the EMG recording showed burst activity of the bilateral buccinator muscle with a duration of 100 to 170 ms, which lasted 5 to 20 s.

**Fig. 2:**
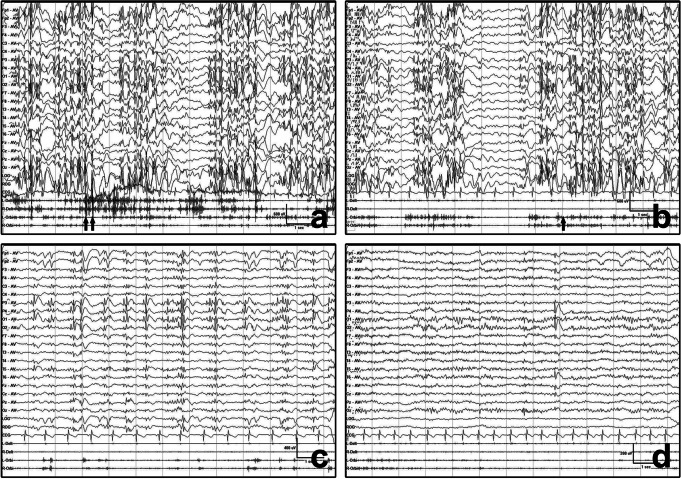
Example of electroclinical characteristics of Patient 1 with SE before and after administration of medication. **a** EEG showed polyspike/spike-wave discharge activity associated with myoclonic absence status, EMG records showed occasional burst activity of the bilateral deltoid muscle associated with paroxysmal upper limb jerk (indicated by arrows). **b** Start of oral administration of 5 mg of midazolam (indicated by arrow). **c** The EEG discharges attenuated and eyelid fluttering improved after 10 min of midazolam administration. **d** EEG activity returned to baseline and the myoclonic seizures terminated after 25 min of midazolam administration. L-Delt: left deltoid, R-Delt: right deltoid, L-Orbi: left orbicularis oculi, R-Orbi: right orbicularis oculi

SE in four patients identified by physicians was treated with oral administration of 5 mg midazolam hydrochloride during EEG monitoring, including three patients (Patients 1, 3 and 4) with eyelid myoclonic absence status and one patient with myoclonic SE (Patient 7). In patients 1 and 4, oral midazolam was given 30 and 60 min after SE initiation, respectively. The clinical seizures and EEG discharges in the two patients improved 2 and 10 min, respectively, after medication, and returned to baseline at 3 and 25 min, respectively (Fig. 2). For Patient 3, oral midazolam was given 13 min after SE initiation, and the ED activity gradually returned to baseline 30 min after administration of midazolam, but intermittent generalized slow waves persisted. In the patient with myoclonic SE (Patient 7), oral midazolam was given 11 min after SE initiation, and the myoclonic jerks and EDs were significantly relieved 5 min later. The continuous background activity reoccurred, but the episode jerks terminated 30 min later. In contrast, SE in the remaining five patients was not identified and treated in time. In two cases of myoclonic SE and one case of eyelid myoclonic absence status, SE eventually terminated with a spontaneous tonic-clonic seizure. All patients were prescribed broad-spectrum antiseizure medications (ASMs) after EEG monitoring, and SE did not recur in any of the patients. Additionally, seven patients reported seizure freedom for a period ranging from 3 months to 6 years.

## Discussion

SE frequently occurs in patients with critical brain disease or systemic disease; however, it has rarely been reported in patients with GGE in Chinese population. In this study, we report a series of nine patients with GGE who presented with different electroclinical features of SE, involving myoclonic absence status, myoclonic SE and autonomic SE. In line with previous evidence [11], we found that females were more prone to SE than males, which may be due to the less use of valproate in females. Compared to the reported median age of 38.6 [12], we found a younger age (16.8 years) at SE occurrence. This discrepancy in the median age of SE onset may be due to the incorrect diagnosis, ASM regimen, and a phenomenon of SE occurring in younger patients with GGE in China.

SE is defined by ILAE as a condition resulting either from the failure of the mechanisms responsible for seizure termination or from the initiation of mechanisms that lead to abnormally prolonged seizures after time point t1. In general, the time point t1 refers to the time point at which treatment is considered to be initiated [13], which is 5 min for generalized tonic-clonic seizures, 10 min for focal seizures with or without impairment of consciousness, and 10–15 min for absence SE after seizure onset [4]. However, a clear definition of t1 is lacking for myoclonic absence status, myoclonic SE and autonomic SE. The durations of myoclonic SE, eyelid myoclonic absence status, autonomic SE, and perioral myoclonic absence SE in the patients of our study were 24 to 210 min, 16 to 350 min, 15 to19 min, and 20 s, respectively, indicating that SE duration dramatically varied across different seizure subtypes. In view of the impact of absence on patients, we believe that the t1 time for eyelid myoclonic absence status can also be determined based on the absence duration state. Oğuz-Akarsu et al. defined myoclonic SE as myoclonia lasting for 30 min or longer without any interrupting non-ictal periods of >3 min in patients with JME [14]. As for perioral myoclonic SE, the episodes lasting 20 s was significantly longer than the common duration (2 to 9 s), so it was regarded as SE.

Several studies have suggested that SE is more likely to occur in patients with juvenile absence epilepsy compared to other GGEs, and that absence SE and myoclonic SE are the most common types of SE in GGE [10, 12]. However, in our study, ELMA and myoclonic absence status were the most commonly detected SE types. This discrepancy may be due to the failure to distinguish between the absence SE and ELMA in different reports. Autonomic SE has been reported with nausea, vomiting, and deviation of the eyes with or without impaired awareness, and usually occurs in patients with Panayiotopoulos syndrome [15–17]. To our knowledge, autonomic SE presenting with a change of heart rate has not been identified in prior study of ELMA. In Patient 2, autonomic SE developed with normal consciousness during EEG monitoring, and it was not found until a technologist reviewed the EEG. The autonomic SE is often misdiagnosed, sometimes even undetected because of varying symptoms, so real-time review of EEG is critical to detect SE and initiate medication for SE. Interestingly, in our study, myoclonic SE and myoclonic absence status were the most common SE types in JME and ELMA, respectively, which indicated that the seizure type of SE occurring in GGE patients is consistent with the main seizure type of their epilepsy syndrome. The different subtypes of SE in patients with GGE may be related to the unique epileptogenic brain network underlying the epilepsy syndrome.

SE is an emergency which requires immediate and effective treatment as timely as possible to reduce neuronal damage, morbidity, and mortality [2, 3]. Evidence supports the view that intravenous administration of benzodiazepines is preferred for emergent initial treatment [18–21]; however, benzodiazepine treatment may increase the risk of respiratory and circulatory inhibition, infection, and death [22]. Rossetti et al. recommended that SE patients with prior epilepsy syndromes often recover well and should be treated with increased doses of the original ASMs [23]. Similarly, in our study, SE in patients with GGE terminated spontaneously or following oral administration of small doses of midazolam, with the latter not been reported in prior studies. As such, aggressive management may not be necessary for SE in patients with GGE, considering the possible risk of respiratory issues and cardiac arrest associated with intravenous administration of benzodiazepines.

There are some limitations in this study. First, retrospective studies may omit some cases of SE, especially those presenting with nonconvulsive status epilepticus. Second, the cases in our study were from the EEG monitoring center of the neurology department, while cases from pediatrics, intensive care units, and the emergency department were lacking. Finally, the number of patients enrolled in this study is small, and further large-sample clinical studies are needed to clarify the electroclinical characteristics of SE in patients with GGE in the Chinese population.

## Conclusions

The results of the current study suggest that the seizure type of SE in patients with GGE is often consistent with their major symptoms, which may be related to different epileptogenic brain network underlying the epilepsy syndrome. In addition, episodes of SE may be an easily treatable complication of GGEs, and could be terminated by oral midazolam.

## Data Availability

The data that support the findings of this study are available from the corresponding author upon reasonable request.

## Abbreviations

ASMs: Antiseizure medications
ELMA: Epilepsy of eyelid myoclonia with absences
EDs: Epileptiform discharges
GGE: Genetic generalized epilepsy
IGE: Idiopathic generalized epilepsy
JME: Juvenile myoclonic epilepsy
MRI: Magnetic resonance imaging
POMA: Perioral myoclonia with absences
SE: Status epilepticus
